# Chemokines induce axon outgrowth downstream of Hepatocyte Growth Factor and TCF/β-catenin signaling

**DOI:** 10.3389/fncel.2013.00052

**Published:** 2013-04-30

**Authors:** Deepshikha Bhardwaj, Mireia Náger, Judith Camats, Monica David, Alberto Benguria, Ana Dopazo, Carles Cantí, Judit Herreros

**Affiliations:** ^1^Depatments of Basic Medical Science and Experimental Medicine, IRBLleida-University of LleidaLleida, Spain; ^2^Genomic Unit, CNICMadrid, Spain

**Keywords:** beta-catenin, axon, neurite outgrowth, chemokine, hippocampal neurons, hepatocyte growth factor

## Abstract

Axon morphogenesis is a complex process regulated by a variety of secreted molecules, including morphogens and growth factors, resulting in the establishment of the neuronal circuitry. Our previous work demonstrated that growth factors [Neurotrophins (NT) and Hepatocyte Growth Factor (HGF)] signal through β-catenin during axon morphogenesis. HGF signaling promotes axon outgrowth and branching by inducing β-catenin phosphorylation at Y142 and transcriptional regulation of T-Cell Factor (TCF) target genes. Here, we asked which genes are regulated by HGF signaling during axon morphogenesis. An array screening indicated that HGF signaling elevates the expression of chemokines of the CC and CXC families. In line with this, CCL7, CCL20, and CXCL2 significantly increase axon outgrowth in hippocampal neurons. Experiments using blocking antibodies and chemokine receptor antagonists demonstrate that chemokines act downstream of HGF signaling during axon morphogenesis. In addition, qPCR data demonstrates that CXCL2 and CCL5 expression is stimulated by HGF through Met/b-catenin/TCF pathway. These results identify CC family members and CXCL2 chemokines as novel regulators of axon morphogenesis downstream of HGF signaling.

## Introduction

The establishment of the neuronal morphogenesis is a complex process by which neurons extend and branch out an axon and dendrites, resulting in the proper assembly of the neuronal circuitry. A range of secreted molecules, including growth factors and morphogens, promote axonal, and dendrite outgrowth. Among them, the Neurotrophins (NT), Hepatocyte Growth Factor (HGF), and Wnts regulate neuronal survival, neurite outgrowth, synaptogenesis, and synaptic plasticity (Maina and Klein, 1999; Korhonen et al., 2000; Chao, 2003; Yu and Malenka, 2003; Ciani and Salinas, 2005; Nakano et al., 2007; Park and Shen, 2012). HGF signaling through its tyrosine kinase receptor Met provides neurotrophic signals to hippocampal neurons (Korhonen et al., 2000) and promotes axon outgrowth (David et al., 2008). How neurons interpret this variety of signals to develop unique axon arbor morphologies is just beginning to be understood.

β-catenin, a component of the cell–cell adhesion complex and an effector of canonical Wnt signaling, plays a key role in axon outgrowth, dendritogenesis, and synapse formation (Murase et al., 2002; Bamji et al., 2003, 2006; Yu and Malenka, 2003; Lu et al., 2004). Briefly, canonical Wnt signaling results in the cytosolic stabilization of β-catenin, which in the absence of Wnt is degraded through the ubiquitin-proteasome system (Li et al., 2012). Stabilized β-catenin translocates to the nucleus and together with Lymphoid Enhancer Factor-1 (LEF-1)/T-cell factor (TCF) transcription factors regulates the expression of Wnt target genes (Behrens et al., 1996; Molenaar et al., 1996). On the other hand, β-catenin binding to the adhesion complex components cadherin and α-catenin is altered by β-catenin tyrosine phosphorylation, resulting in the downregulation of cell adhesion and the promotion of migration (Nelson and Nusse, 2004; Heuberger and Birchmeier, 2010). We previously showed a requirement for β-catenin phosphorylation at Y654 and Y142 in the axon outgrowth promoted by the NT and HGF signaling, respectively. HGF signaling induces the phosphorylation of β-catenin at Y142 (PY142), which translocates to the nucleus and promotes axon morphogenesis through TCF4/β-catenin-dependent transcription of target genes (David et al., 2008). These findings highlight the relevance of β-catenin forms producing transcriptional regulation independent of Wnt signaling (Monga et al., 2002; Zeng et al., 2006; Heuberger and Birchmeier, 2010; Xi et al., 2012).

Chemotactic cytokines (“chemokines”) are small proteins classified into four subgroups referred to as CXC/α, CC/β, CX_3_C/δ, or C/δ families (Zlotnik and Yoshie, 2000; Tran and Miller, 2003) according to the position and spacing of cysteine residues important for their tri-dimensional structure. Chemokines are best known for their role in leukocyte migration in host immune surveillance and inflammatory responses. However, chemokines and their G-protein-coupled receptors are also expressed by neurons and glia in the nervous system. Interestingly, chemokines of the CXC and CC families have been implicated in proliferation, neurogenesis, and neuronal differentiation of neural precursors (Tran and Miller, 2003; Edman et al., 2008a,b; Wu et al., 2009). Meningeal CXCl12/Sdf-1 signaling through its receptor CXCR4 regulates the migration of cerebellar progenitors (Zou et al., 1998; Reiss et al., 2002) and Cajal-Retzius cells (Borrell and Marin, 2006). CXCL12 also controls interneuron migration during cortical development (Stumm et al., 2003; Lopez-Bendito et al., 2008; Lysko et al., 2011; Sanchez-Alcaniz et al., 2011; Wang et al., 2011). Furthermore, CXCL12 reduces axon outgrowth and branching in hippocampal neurons (Pujol et al., 2005). Importantly, neuronal migration and morphogenesis are coordinated processes that appear inversely regulated by CXCL12 signaling. CXCL12 increases the rate of interneuron migration while reducing neurite branching, whereas blocking CXCL12 signaling enhances neurite branching, thus explaining how interneurons switch from migratory streams to invade the cortical plate and branch out extensively (Lysko et al., 2011). Moreover, astrocyte-secreted CCL5/Rantes induces the outgrowth of cortical neuron neurites (Chou et al., 2008). CCL5 secretion is suppressed in astrocytes from a Huntington mouse model (Chou et al., 2008), indicating that chemokine signaling is involved in neuronal physiology and pathology.

Here we asked which are the genes regulated by HGF/β-catenin signaling during axon morphogenesis. We observe that expression of CC and CXC chemokines is upregulated by HGF signaling in hippocampal neurons. We find that chemokines promote axon outgrowth in hippocampal neurons, the most remarkable one being CXCL2 that also stimulates axon branching. Experiments using chemokine blocking antibodies and pharmacological inhibitors of chemokine receptors demonstrate that chemokines act downstream of HGF signaling. We also show that chemokine expression is reduced upon Met and TCF inhibition. These results identify the chemokines as novel regulators of axon morphogenesis downstream of HGF and β-catenin/TCF signaling.

## Materials and methods

### Materials

HGF was purchased from Peprotech, Wnt-3a from Millipore, Hoescht-33258, SU11274, and FH535 were from Sigma, and SB225502 and SB324837 from Tocris. Antibodies were purchased from the following companies: βIII-tubulin from Covance, β-actin from Sigma, β-catenin from Becton-Dickinson and anti-rat CCL20 and CXCL2 antibodies from R&D. Rat CCL5, CCL7, CCL20 were from R&D Systems and CXCL2 from Peprotech.

### Hippocampal cultures

Rat primary hippocampal neurons were isolated from 18–19 day embryos and cultured in DMEM medium supplemented with N2 and B27. Neurons were plated on poly-D-lysine coated (500 μg/ml) glass coverslips for immunostaining (40 cells/mm^2^) or on plastic (1000–1500 cells/mm^2^) for RNA isolation.

### Immunofluorescence and axon measurements

Neurons plated on coverslips were treated at the first day in vitro (1DIV) and fixed at 2DIV. Treatments were as follows: 10, 300, or 1000 ng/ml for chemokines; HGF 50 ng/ml; Wnt-3a 100 ng/ml; SU11274 2 μ M; FH535 10 μ M; SB225502 1.25 nM; SB324837 20 nM; blocking antibodies against rat CCL20 and CXCL2 (40 μg/ml) or ovalbumin at the same concentration. Neurons were fixed with 4% paraformaldehyde (PFA) for 20 min at RT. Cells were then washed with phosphate buffer saline (PBS) and blocked and permeabilized in PBS containing 5% Foetal calf serum, 5% Horse serum, 0.2% glycine, and 0.1% Triton X100, before incubation with βIII-tubulin antibody. Secondary antibodies were Alexa Fluor488 or Fluor594 (Molecular Probes). Coverslips were mounted on Mowiol. Micrographs were obtained using an inverted Olympus IX70 microscope (10×, 0.3 NA, or 20×, 0.4 NA) equipped with epifluorescence optics and a camera (Olympus OM-4 Ti). Images were acquired using DPM Manager Software and processed using MacBiophotonics ImageJ software (www.macbiophotonics.ca). Axon length was measured using Adobe Photoshop software and the axon was identified as the longest neurite at this stage (2 DIV) of the hippocampal cell development. Images were inverted using Photoshop and are shown on a white background for a clearer visualization of their morphology. Branching was measured by counting Total Axonal Branch Tip Number (TABTN) (Yu and Malenka, 2003). Typically, 15–20 neurons were measured/condition in ≥ three independent experiments. Axon length and branching plots represent values compared to the corresponding untreated control, shown as average ± s.e.m. Significance was calculated by the Student's *t*-test. Asterisk (^*^) indicates statistical significance compared to the corresponding untreated control and hash (#) compared to stimulated controls (see legends for details).

### Luciferase assay

To determine β-catenin transcriptional activation status, luciferase assay was performed following transfection of the TOP-Flash plasmid that carries a synthetic promoter containing three copies of the TCF-4 binding site upstream of a firefly luciferase reporter gene. Hek293T cells were plated at a density of 100 cells/mm^2^ and transfected with Lipofectamine 2000 (Life Technologies) on the day next after plating. Treatments were given on the following day for 24 h (HGF 50 ng/ml; Wnt-3a 100 ng/ml; FH535 8 μM and SU11274 2 μM). After 48 h of transfection, cells were lysed in lysis buffer 25 mM glycylglycine, pH 7.8, 15 mM Mg_2_SO4, 1% Triton X-100, 5 mM EGTA and rocket on ice for 15 min. Luciferase activity in the cell lysates was determined in Luciferase Buffer (25 mM glycylglycine, 15 mM KHPO_4_, pH 7,8, 15 mM Mg_2_SO_4_, 1% Triton X-100, 5 mM EGTA, 1 mM dithiothreitol, 2 mM ATP, 100 mM acetyl-coenzymeA, and 100 mM luciferine) using a microplate luminometer. Luciferase activity was normalized for the total protein concentration in each condition.

### shRNA expression by lentiviral infection

shRNA vectors specific for rat β-catenin were transfected into Hek293T cells together with the plasmids psPAX2 and pMD2G, as previously described (David et al., 2008). The Hek293T medium was collected after 48 h of transfection and centrifuged at 50,000×g for 3 h. The viral pellet was re-suspended in sterile PBS plus 2% Bovine Serum Albumin. Hippocampal neurons were transduced 3–4 h after plating. According to GFP expression driven by the lentiviral vector ~90% of neurons were transduced. Neurons were treated with HGF alone or together with pharmacological inhibitors at 3 DIV and collected at 4 DIV for mRNA purification (RNA isolation kit; Macherey-Nagel). Efficiency of the silencing induced by shRNA was evaluated by Western-blotting of 4DIV hippocampal neuron cell lysates. Densitometric analysis of the bands was performed using Scion software and β-catenin levels were normalized to the intensity of β-actin band.

### RNA isolation

For RNA isolation, treatments were performed as for immunofluorescence studies. Pervanadate was applied for the last 2 h of HGF stimulation. RNA was isolated using Nucleospin RNA II kit (Macherey-Nagel), including a DNase digestion step to remove contaminant DNA.

### Array processing and array data analysis

RNA samples (800 ng) were amplified and labeled with Cy3-CTP using the One-Color Microarray-Based Gene Expression Analysis Protocol (Agilent Technologies, Palo Alto, CA, USA) and hybridized to Whole Rat Genome Microarray 4 × 44K (G4131F, Agilent Technologies).

Raw data files from the scanned arrays were extracted using Feature Extraction software version 9 (Agilent Technologies). Data files from Feature Extraction software were imported into GeneSpring® GX software version 9.0. (Agilent Technologies). Quantile normalization was performed (Bolstad et al., 2003) and expression values (log2 transformed) were obtained for each probe. Probes were also flagged (*Present*, *Marginal*, *Absent*) using GeneSpring® default settings. Probes with signal values above the lower percentile (20th) and flagged as *Present* or *Marginal* in 100% of replicates in at least one out of the two conditions under study, were selected for further analysis. Paired *t*-test was performed between conditions to be tested for differential expression analysis. Raw *p*-values were corrected for false discovery rate control using Benjamini–Hochsberg's method (Benjamini and Hochberg, 1995).

### Reverse-transcriptase PCR

mRNA was reverse-transcribed (RT) to cDNA (25°C for 10 min, 42°C for 60 min, and 95°C for 5 min) using random hexamers and Superscript II reverse transcriptase (Applied Biosystems). Negative control RT-minus reactions were carried out to confirm absence of DNA contamination in RNA.

### Semi-quantitative PCR

To detect the relative expression of different chemokine genes in HGF-treated and untreated samples, semi-quantitative (sq) PCR was run. Equal volumes of cDNA were amplified by PCR using a couple of specific primers expanding at least two exons within the gene of interest. Sequences of the primers used were: CCL5 forward atatggctcggacaccactc, CCL5 reverse cccacttcttctctgggttg, CCL7 forward gggaccaattcatccacttg, CCL7 reverse cctcctcaacccacttctga, CCL20 forward gcttacctctgcagccagtc, CCL20 reverse cggatcttttcgacttcagg, CXCL2 forward agggtacaggggttgttgtg, CXCL2 reverse tttggacgatcctctgaacc. Ten microlitre aliquots taken from 25, 30, and 35 PCR cycles (CXCL2 and CCL7), 30, 34, and 38 PCR cycles (CCL5) or 24 and 28 PCR cycles (CCL20) were analyzed in 3% agarose gel. Densitometry of the DNA bands was performed using the Scion Image software (Scion Corporation) and comparing measurements from non-saturated PCR products. Loading was checked by amplification of the GAPDH transcript. Transcript analysis was performed from at least three independent simples.

### Real time PCR (qPCR)

cDNA processed from 1 μg RNA was used as the template. One microlitre aliquot of each cDNA was used per well. Samples were run in triplicate. Expression of the transcript levels were analysed using a FAM-labeled CXCL2 or CCL5 probes and compared to that of GAPDH, used as a loading control, in a ABI Prism 7000 HT sequence detection system (Applied Biosystems). Relative expression was calculated using the ΔΔC1 method.

## Results

### Chemokines of the CC and CXC families are upregulated by HGF treatment

**Figure 1:**
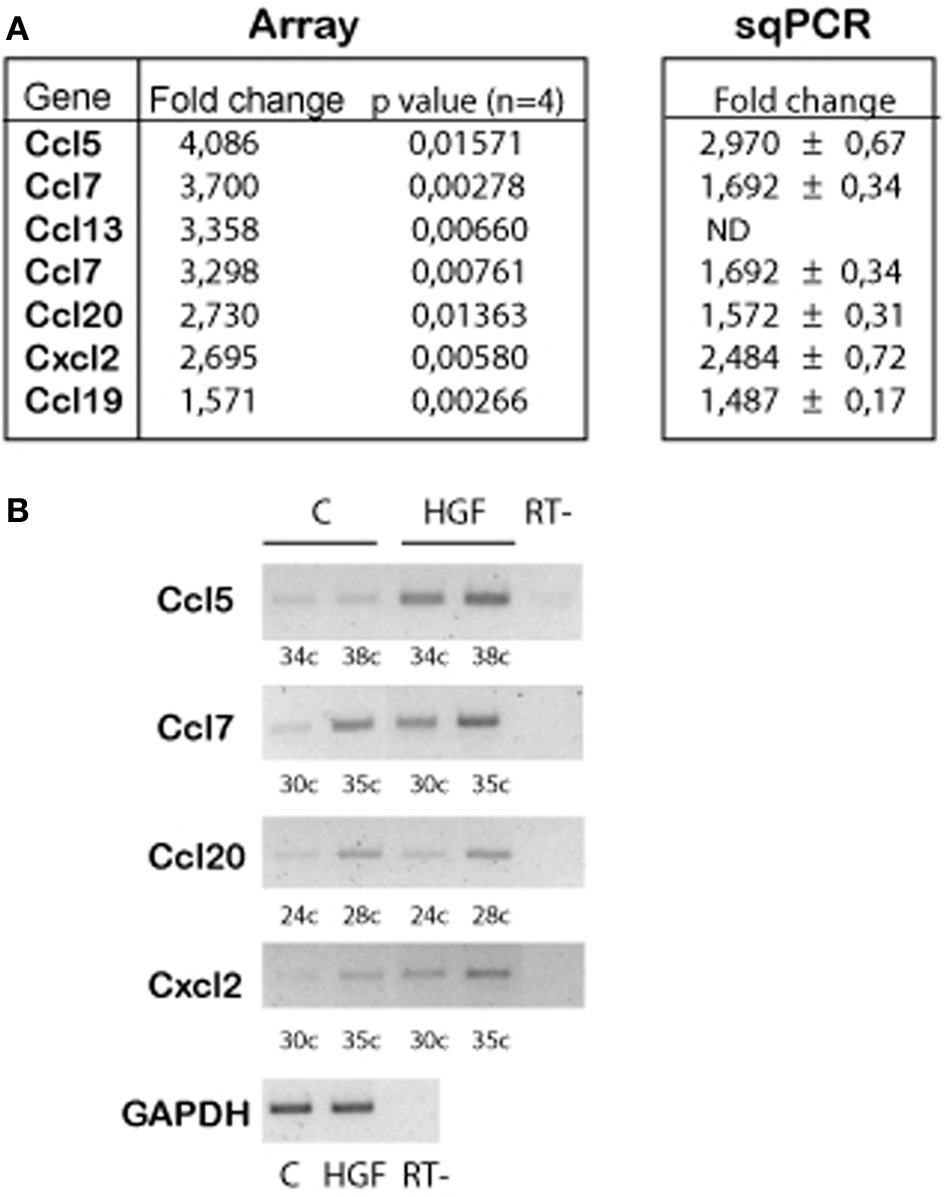
**Chemokine genes are upregulated by HGF signaling in 2DIV hippocampal neurons. (A)** Summarized array data (left) indicating the chemokine genes that are upregulated in HGF-treated (50 ng/ml, 24 h) compared to untreated hippocampal neurons. (Right) Summary of the quantification of sqPCR experiments. Values indicate fold change of the chemokine expression in HGF-treated vs. untreated samples ± s.e.m. (≥3 experiments). **(B)** Representative sqPCR of samples taken at the indicated PCR cycle to compare the expression of chemokines in untreated and HGF-treated hippocampal neurons. GAPDH was used as a housekeeping gene (image corresponds to 30 PCR cycles). RT-indicates samples in which reaction was run without RT enzyme.

Previous work demonstrated that HGF signals through PY142 β-catenin and TCF4 to regulate the expression of target genes during hippocampal neuron development (David et al., 2008). We questioned which are the genes regulated upon HGF stimulation in axon morphogenesis. We performed array experiments using control and HGF-treated hippocampal neuron samples. Array results revealed an upregulation of several chemokine genes in HGF-treated neurons compared to untreated neurons (Figure 1A), which was confirmed by sqRT-PCR (Figures 1A,B). *In silico* analysis of the 2 kb region upstream of the ATG in the identified chemokine genes showed the presence of several copies of putative TCF-binding sites, as predicted for β-catenin/TCF-target genes (data not shown). These findings indicated that chemokines may be involved in the HGF-induced axon morphogenesis.

### Chemokine signaling promotes axon morphogenesis

**Figure 2:**
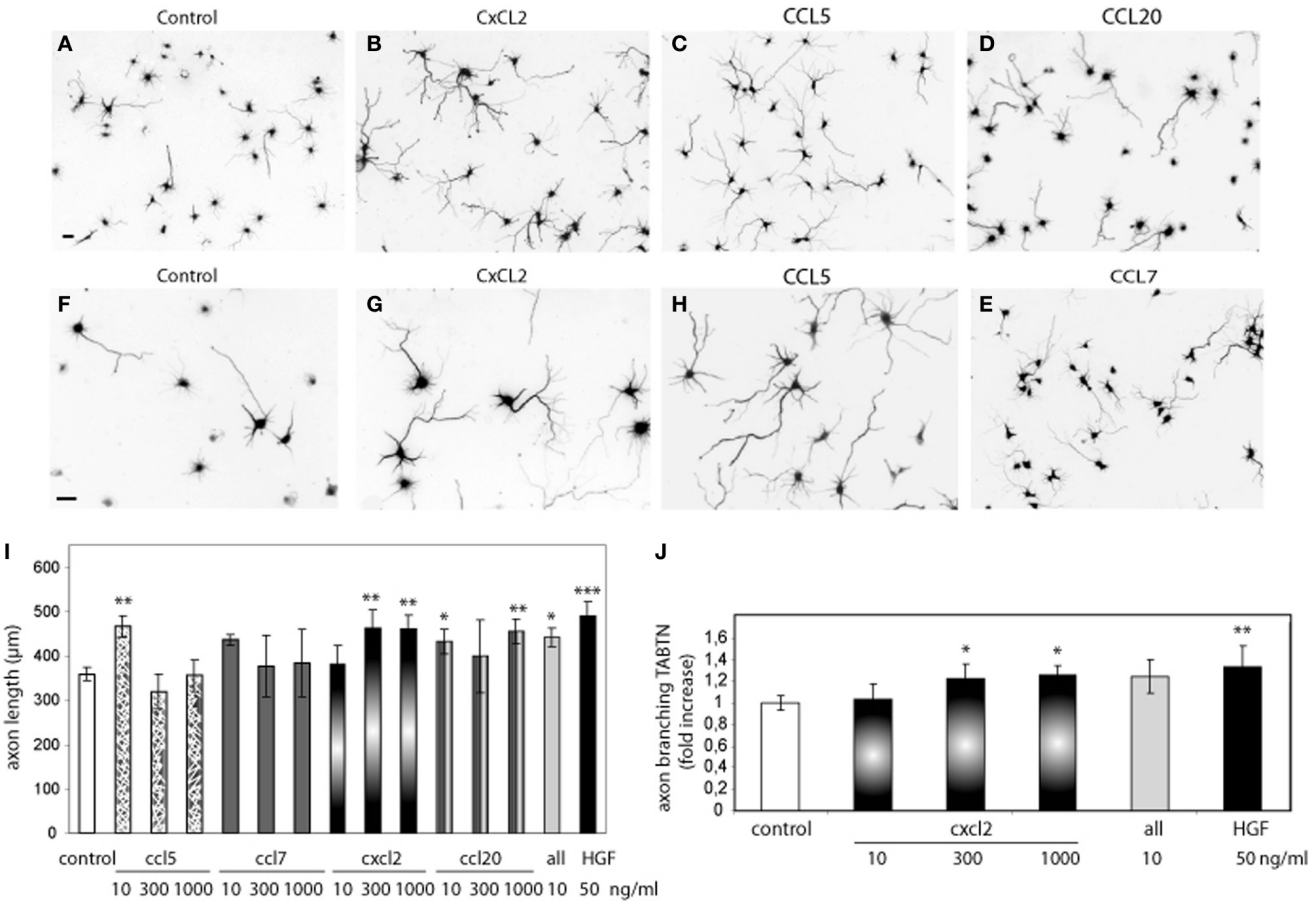
**Recombinant chemokines increase axon morphogenesis. (A–H)** Hippocampal neurons (2 DIV) control or treated with CXCL2, CCL5, CCL20, and CCL7 (1000 ng/ml) and immunostained for βIII-tubulin to reveal the axon morphology. Images **(A–E)** were taken at 10× and **(F–H)** at 20×. Bars = 30 μm. Average axon length compared to control **(I)** and axon branching shown as an increase vs. control **(J)** for chemokine treatments at the indicated dose or HGF (50 ng/ml). *All* refers to a cocktail of the four chemokines (10 ng/ml). ^*^p ≤ 0.05, ^**^p ≤ 0.01, and ^***^p ≤ 0.001.

To address this possibility, we first tested whether chemokines induce axon outgrowth and branching. Hippocampal neurons were treated with CCL5, CCL7, CCL20, or CXCL2 at different concentrations (10–1000 ng/ml). CCL5 (10 ng/ml), CXCL2 (300 and 1000 ng/ml), and CCL20 (10 and 1000 ng/ml) significantly increased the total length of the axon compared to axon length values of untreated neurons (Figure 2). A cocktail of all the chemokines (10 ng/ml) also increased axon outgrowth (Figure 2I). The increases in axon length were in the range of that obtained by HGF stimulation (Figure 2I). In addition to increasing axon length, CXCL2 also produced axon branching (Figure 2J). Axon branching was not significant for the other studied chemokines at the tested concentrations (data not shown).

**Figure 3:**
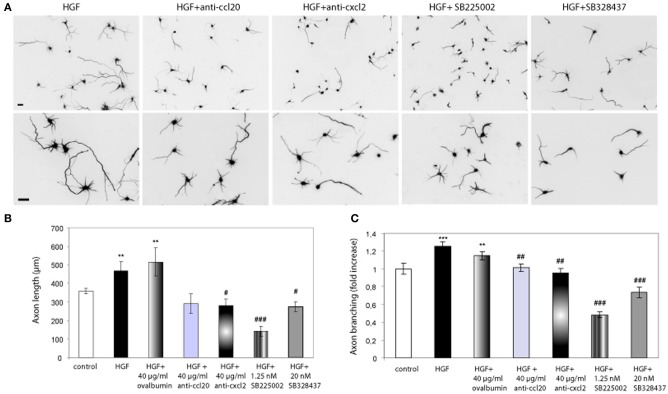
**Chemokine signaling is involved in the axon morphogenesis promoted by HGF. (A)** Hippocampal neurons treated with HGF (50 ng/ml) together with anti-CCL20 (40 ug/ml), anti-CXCL2 (40 μg/ml), SB225502 (1.25 nM) or SB324837 (20 nM), and immunostained for βIII-tubulin. Top pictures were taken at 10× and bottom at 20×. Bars = 30 μm. **(B** and **C)** Average axon length measurements **(B)** and axon branching shown as an increase vs. control **(C)**. *All* refers to a cocktail of the four chemokines (10 ng/ml). ^**^p ≤ 0.01 and ^***^p ≤ 0.001 when comparing to control neurons. #*p* ≤ 0.05, ##*p* ≤ 0.01, and ###*p* ≤ 0.001 when comparing to HGF-treated neurons.

Having showed that exogenously added chemokines induce axon morphogenesis in hippocampal neurons, we studied whether blocking chemokine signaling would inhibit the effect of HGF on axon morphogenesis. To this end, we used blocking antibodies against the chemokines as well as the chemokine receptor antagonists SB2250002 and SB328437 (White et al., 1998, 2000). Neurons incubated with HGF together with antibodies against rat CXCL2 or CCL20 (40 μg/ml) displayed axon length and branching values similar to those of untreated neurons (Figure 3). However, the increase in axon length promoted by HGF was not affected by the presence of ovalbumin at the same concentration than the antibodies (40 μg/ml). Furthermore, treatment with HGF and the antagonist for the receptor of CXCL2 (CXCR2) SB2250002, or with SB328437, an antagonist of CCR3 (that acts as the only receptor of CCL20 and one of the receptors of CCL5), potently inhibited axon outgrowth and branching to values below those of control neurons (Figure 3). These results suggest that CXCL2 and CCL20 are secreted upon HGF stimulation and that endogenous CXCL2 and CCL20 signaling plays a role in axon morphogenesis.

### HGF regulates CXCL2 expression through met and β-catenin/TCF

**Figure 4:**
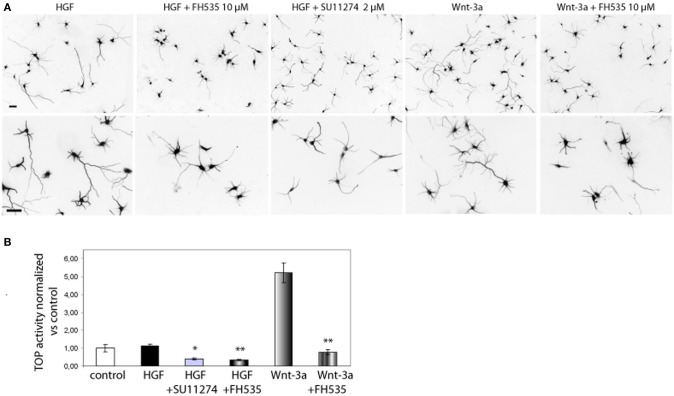
**Met and TCF inhibition reduce the axon outgrowth induced by HGF. (A)** Hippocampal neurons treated with HGF (50 ng/ml) or Wnt-3a (100 ng/ml) alone or together with FH535 (10 μ M) or SU11274 (2 μ M) were immunostained for βIII-tubulin to reveal the axon morphology. Top pictures were taken at 10× and bottom at 20×. Bars = 30 μm. **(B)** β-catenin transcriptional activity determined using the TOP reported plasmid in transfected Hek293 cells. Relative luciferase activity was determined at 48 h after transfection. Treatments were applied for the last 24 h (see Materials and Methods for details). ^*^p ≤ 0.05, ^**^p ≤ 0.01.

We sought to study the pathway regulating chemokine expression downstream of HGF signaling. We demonstrated that HGF stimulation induces PY142 β-catenin phoshorylation and transcriptional regulation by TCF/β-catenin (David et al., 2008). To check if chemokine expression is controlled by TCF and β-catenin downstream of HGF signaling, we followed both a pharmacological and a gene silencing approach. We used SU11274 that inhibits Met activity (Berthou et al., 2004) and FH535, which inhibits TCF/β-catenin by blocking the recruitment of β-catenin to the promoter of target genes (Handeli and Simon, 2008). First, we studied β-catenin transcriptional activation using the TOP reporter plasmid in Hek293 cells, in which HGF/Met signaling is active (Royal and Park, 1995). Although HGF did not stimulate luciferase reporter activity vs. control, treatment with SU11274 significantly reduced β-catenin transcriptional activation (Figure 4B). This result suggests that in Hek293 cells, HGF/Met signaling through β-catenin is already active in basal conditions, likely by an autocrine production of HGF. Furthermore, treatment with FH535 significantly reduced luciferase activity, demonstrating that TCF activation is involved in HGF/Met signaling (Figure 4B). We used Wnt-3a as a positive control, which produced a clear activation of luciferase activity and was also inhibited by FH535 (Figure 4B). These results confirmed FH535 as an effective TCF/β-catenin inhibitor and indicated that HGF/Met signal through TCF/β-catenin in Hek293 cells among other cell systems (Monga et al., 2002).

In hippocampal neurons, both SU11274 and FH535 inhibited the axon outgrowth induced by HGF signaling (Figure 4A). As a positive control we also treated neurons with Wnt-3a with or without FH535. As expected, TCF inhibitor blocked the axon outgrowth promoted by Wnt-3a. FH535 treatment also blocked the axon outgrowth promoted by HGF, rendering axon length values below those of control neurons, thus confirming that HGF signaling is dependent on TCF-driven transcription (David et al., 2008).

**Figure 5:**
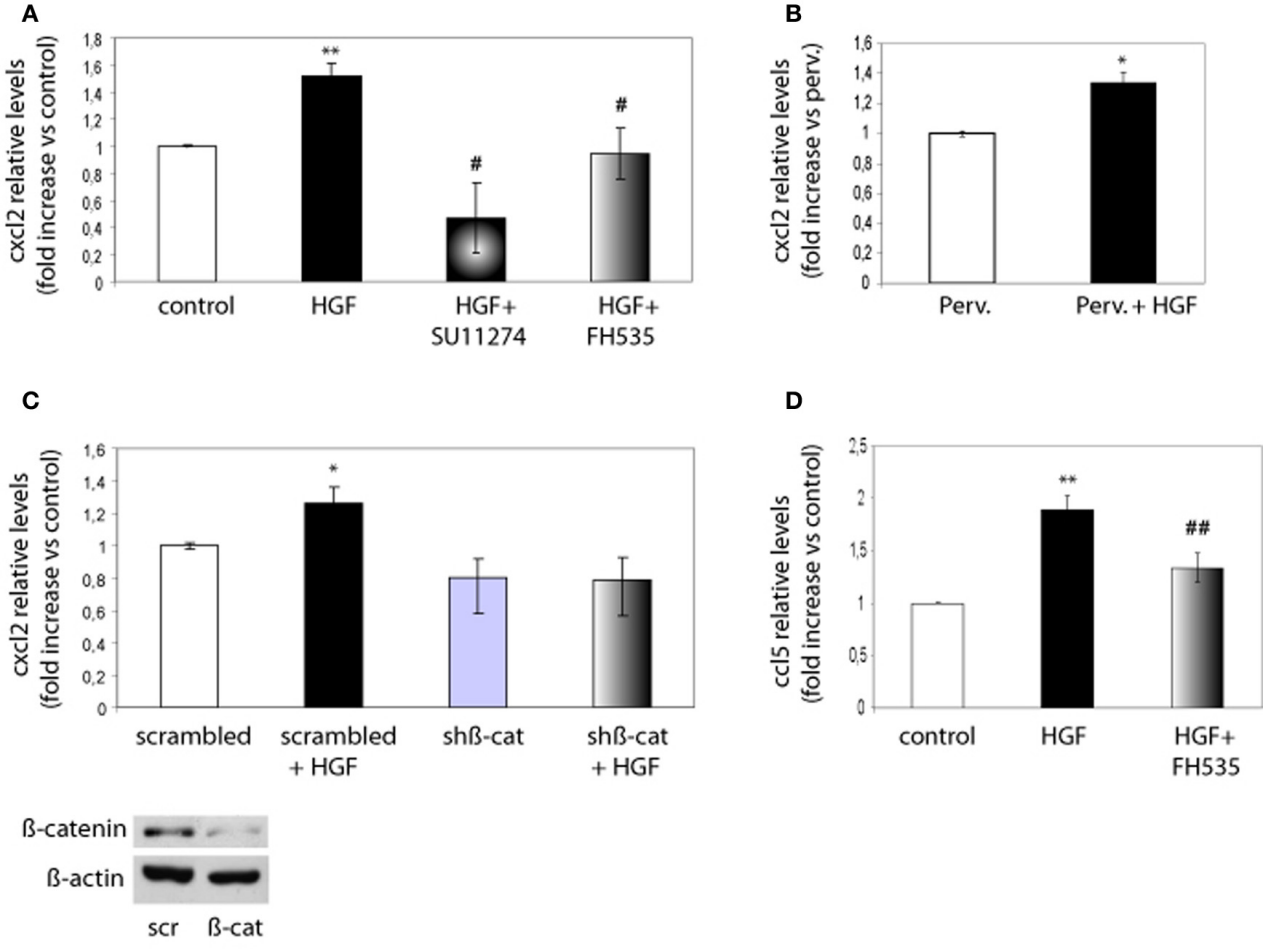
**CXCL2 and CCL5 expression is regulated by HGF signaling through TCF/β-catenin. (A)** qPCR analysis indicates that CXCL2 relative levels in 2 DIV hippocampal neurons increase upon HGF stimulation (50 ng/ml, 24 h), decrease below control levels in neurons co-treated with SU11274 (2 μM) plus HGF and remain similar to control levels in neurons co-treated with FH535 (10 μM) plus HGF. **(B)** CXCL2 relative levels upon HGF stimulation in pervanadate (perv)-treated (last 2 h) neurons. **(C)** CXCL2 relative levels in 4 DIV hippocampal neurons transduced with lentiviral vectors driving the expression of scrambled or β-catenin shRNAs, untreated or treated with HGF stimulation, indicate that HGF does not increase CXCL2 levels in neurons expressing β-catenin shRNA. Lower panel: Western-blot for β-catenin shows a reduction in β-catenin levels in hippocampal neurons expressing scrambled (scr) or β-catenin (β-cat) shRNAs. β-actin was used as a loading control. **(D)** CCL5 relative levels (4 DIV) increase by HGF and significantly decrease when neurons were co-treated with HGF and FH535. ^*^p ≤ 0.05, ^**^p ≤ 0.01 when comparing to control neurons. #*p* ≤ 0.05, ##*p* ≤ 0.01 when comparing to HGF-treated neurons.

Next, we investigated whether chemokine expression is regulated through the HGF/Met/TCF/β-catenin pathway. Because CXCL2 promoted both axon outgrowth and branching, we focused on this chemokine and on CCL5 as a member of the CC family. We analyzed the expression of these chemokines using real time qPCR from control neurons, neurons treated with HGF or treated with HGF together with SU11274 or FH535. Expression of CXCL2 increased by 1.6-fold in HGF-treated neurons compared to untreated neurons, but was reduced to values below those of untreated neurons following the treatment with HGF and SU11274 (Figure 5A). Pervanadate (a tyrosine phosphatase inhibitor) was previously used to stabilize the PY142-β-catenin form (David et al., 2008). However, HGF stimulation increased CXCL2 expression in pervanadate-treated neurons in a similar way than in the absence of pervanadate (Figure 5B). Furthermore, the increase in CXCL2 expression induced by HGF signaling was lost upon the co-treatment of HGF and FH535 (Figure 5A), indicating that it was mediated by TCF/β-catenin. In addition, we analyzed CXCL2 mRNA levels in hippocampal neurons in which β-catenin was silenced following lentiviral-driven shRNA expression. The silencing efficiency obtained with this β-catenin shRNA at four days was around 40–50% as similarly reported (David et al., 2008, 2010). Whereas HGF was able to increase the expression of CXCL2 in neurons expressing scrambled shRNA, this increase was lost in neurons expressing β-catenin shRNA (Figure 5C). We also analyzed the expression of CCL5, which increased nearly 2-fold upon HGF stimulation and was significantly reduced in the presence of HGF and FH535 (Figure 5D).

## Discussion

Aiming to identify the genes regulated by HGF signaling in developing hippocampal neurons during axon morphogenesis, we found the upregulation of several chemokines of the CC and CXC families. Following array experiments, we demonstrated that CCL5, CCL20, and CXCL2 significantly promote axon outgrowth and, in the case of CXCL2, also axon branching. PCR data confirmed that chemokines are upregulated by HGF signaling. By blocking chemokine signaling, we demonstrated that CCL20 and CXCL2 act downstream of HGF signaling in axon outgrowth and branching. During the establishment of the axon arbor morphology, HGF signaling induces β-catenin PY142 and TCF4-dependent transcriptional regulation of target genes (David et al., 2008). We inquired whether chemokines are regulated through TCF/β-catenin downstream of HGF signaling. TCF inhibition or β-catenin silencing reduced CXCL2 and CCL5 expression upon HGF stimulation. We conclude that chemokines are new molecules modulating axon outgrowth in hippocampal neurons, which expression is regulated by HGF through TCF/β-catenin signaling.

### Chemokine signaling in neuronal migration and axon outgrowth

Chemokines are well-established chemotactic molecules inducing the migration of leukocytes and hematopoietic progenitors (Rossi and Zlotnik, 2000). In the nervous system, chemokines and chemokine receptor expression are regulated under a variety of conditions, including brain repair (Babcock et al., 2003; Miller et al., 2008; Jaerve et al., 2012). Thus, chemokine signaling has been involved in neuroinflammation, the pathogenesis of chronic pain (White et al., 2005), myelination (Kury et al., 2002) and human immunodeficiency virus-1 (HIV-1)-associated neuropathology (Tran and Miller, 2003). Furthermore, a role for chemokines (in particular for CXCL12/Sdf-1) in regulating axon outgrowth and guidance has also been described during nervous system development (Tran and Miller, 2003). CXCL12-CXCR4 signaling is involved in the guidance of motoneuron's axon (Lieberam et al., 2005) and downstream of Sonic Hedgehog signaling in retinal ganglion cell axon pathfinding (Stacher Horndli and Chien, 2012). CXCL12 can either produce growth cone repulsion or attraction depending on the levels of cGMP (Xiang et al., 2002). Remarkably, CXCL12 signaling regulates the migration of different neuron and neuronal progenitor populations: gonadotropin-releasing hormone-1 neurons emerging from the nasal placode (Casoni et al., 2012), interneurons moving from the medial ganglionic eminence toward the cortical plate (Lopez-Bendito et al., 2008; Lysko et al., 2011), cerebellar progenitors (Zou et al., 1998; Vilz et al., 2005) and sensory neuron progenitors toward dorsal root ganglia (Belmadani et al., 2005). CCL2, CCL7, and their receptors are expressed during midbrain development, promoting the differentiation of dopaminergic neurons and also neuritogenesis (Edman et al., 2008a,b). To our knowledge, this is the first work reporting that CC chemokines induce axon morphogenesis in hippocampal neurons. In agreement with our data, *in situ* hybridization data freely available online reveals the expression of chemokines in the mouse hippocampus during embryonic and adult life (see Genepaint and Allen Brain Atlas webpages). CXCL12 was shown to promote the extension of perforant fibers from the entorhinal cortex to dentate gyrus neurons and the migration of dentate granule cells during hippocampal development (Bagri et al., 2002; Lu et al., 2002; Ohshima et al., 2008). In addition, CXCL12 reduces axon elongation while promoting axon branching in dissociated hippocampal neurons (Pujol et al., 2005). We found that CXCL2 increases total axon length and axon branching in hippocampal neurons, suggesting that it plays a role *in vivo* during hippocampal development. In line with an effect of chemokines inducing axon development, mice lacking chemokine receptors showed impairments in hippocampal cognitive function and synaptic plasticity (Rogers et al., 2011; Belarbi et al., 2013).

### HGF/β-catenin signaling and chemokines

Our findings establish a relationship between HGF and chemokine signaling in hippocampal neurons. HGF-treated neurons displayed increased expression of chemokines. Antibodies against CCL20 and CXCL2, as well as the CCR3 and CXCR2 antagonists SB22502 and SB328437, inhibited the axon outgrowth and branching promoted by HGF, implying that chemokines are downstream of HGF signaling. Moreover, treatment with SB22502 or SB328437 resulted in axon length and branching values clearly below those of untreated neurons, suggesting that endogenous chemokine production by hippocampal neurons impacts on axon development. Furthermore, affecting CCL20 signaling by anti-CCL20 antibodies or SB328437 treatment reduced axon branching in the presence of HGF. However, CCL20 at the tested concentrations (10–1000 ng/ml) did not promote significant axon branching. These findings suggest that promotion or inhibition of axon branching exhibit different EC50/IC50 values. Alternatively, CCL20 concentrations lower than tested may induce axon branching.

β-catenin is a classical effector of Wnt signaling and a transcriptional coactivator of LEF/TCF. We and others have described the interaction between Met and β-catenin (Monga et al., 2002; Zeng et al., 2006; David et al., 2008), which results in β-catenin phosphorylation at Y142 *in vitro* (David et al., 2008). In hippocampal neurons, HGF signaling increases PY142-β-catenin, which moves to the nucleus and regulates axonal morphogenesis through TCF4-transcriptional activation (David et al., 2008). Here, we confirm that HGF/Met signaling supports axon outgrowth through TCF/β-catenin transcriptional activity. In line with these findings, the phosphorylation of β-catenin and α-catenin downstream of tyrosine kinase receptor and/or src activation is emerging as a Wnt-independent pathway that promotes β-catenin transcriptional activation and migration of cancer cells (Ji et al., 2009; Xi et al., 2012). In this work chemokines were identified as transcriptional targets of HGF in developing in hippocampal neurons. CXCL2 and CCL5 expression analysis confirmed that these chemokines are regulated by HGF signaling. In addition, TCF inhibition and β-catenin silencing blocked the upregulation of CXCL2 by HGF. A previous paper (Halleskog et al., 2011) described the upregulation of cytokines and chemokines—including CXCL2—by Wnt-3a and β-catenin signaling in activated microglia. TCF inhibition also reduced the expression of CCL5 following HGF stimulation (by ~30% compared to HGF-treated neurons), suggesting that CCL5 is at least in part regulated through TCF/β-catenin downstream of HGF signaling. It is possible that HGF signaling activates another pathway, i.e., NFkB pathway that affects CCL5 expression (Chou et al., 2008).

In summary these findings add different chemokines to the growing list of secreted molecules that modulate axon outgrowth, and highlights new developmental roles for signaling molecules known to regulate immune cell biology. As chemokines play a role in post-ischemic brain repair (Wang et al., 2012) and recruiting stem cells after spinal cord injury (Jaerve et al., 2012), it is tempting to speculate that chemokines at the injury site may serve to improve axon regeneration.

### Conflict of interest statement

The authors declare that the research was conducted in the absence of any commercial or financial relationships that could be construed as a potential conflict of interest.
